# Biochemical Signatures of L-Carnitine-Induced Changes in Brain Cancer Cells Revealed by Confocal Raman Imaging: A Preliminary Study

**DOI:** 10.3390/s26123830

**Published:** 2026-06-16

**Authors:** Jakub Maciej Surmacki, Krzysztof Sergot, Monika Kopeć

**Affiliations:** Laboratory of Laser Molecular Spectroscopy, Faculty of Chemistry, Institute of Applied Radiation Chemistry, Lodz University of Technology, Wroblewskiego 15, 93-590 Lodz, Poland; 250919@edu.p.lodz.pl (K.S.); monika.kopec@p.lodz.pl (M.K.)

**Keywords:** Raman spectroscopy and imaging, brain cancer, astrocytoma, L-carnitine (tartrate)

## Abstract

**Highlights:**

- Raman spectroscopy revealed distinct L-carnitine (tartrate)-induced biochemical alterations in human astrocytoma cells.
- Single-cell chemometric analysis demonstrated treatment-dependent heterogeneity in lipid, protein, and nucleic acid profiles.
- Raman imaging provides a label-free approach for monitoring metabolic responses to L-carnitine (tartrate) supplementation.
- The observed biochemical reprogramming suggests that L-carnitine (tartrate) may modulate cellular metabolism in astrocytoma cells.

**Abstract:**

L-carnitine plays a central role in mitochondrial fatty acid transport and cellular energy regulation; effects on the biochemical phenotype of brain cancer cells remain insufficiently characterized. Here, we applied confocal Raman spectroscopy and imaging to investigate the biochemical alterations induced by L-carnitine supplementation—administered as its tartrate salt—in human astrocytoma cells. Raman spectral analysis revealed distinct changes in lipid-, protein-, nucleic acid-, and cytochrome-associated vibrational features following 24 h of treatment, suggesting alterations in mitochondrial activity and cellular energy-related processes. Principal component analysis identified PC1 (93.87%) as representing the intrinsic biochemical composition of the cells, whereas PC2 (1.19%) and PC3 (0.59%) captured subtle yet consistent variations in lipid organization, protein conformation, and redox-sensitive vibrational features associated with L-carnitine exposure. Pearson correlation analysis of Raman cluster spectra indicated biochemical differences across cellular compartments, with the most pronounced changes observed in lipid droplets, supporting modifications in lipid-associated cellular processes. These findings demonstrate that Raman imaging provides a sensitive, label-free platform for resolving L-carnitine-induced biochemical heterogeneity at the single-cell level. Overall, this study highlights vibrational spectroscopy as a powerful tool for characterizing cellular responses to metabolic modulators and provides insight into the biochemical impact of exogenous L-carnitine in brain cancer cells.

## 1. Introduction

Cancer continues to dominate medical discourse as one of the most lethal diseases worldwide, its mortality driven by delayed detection and the absence of curative treatment. In 2022, around 20 million new cases were diagnosed, with 9.7 million deaths globally [1]. Within this context, we focused on astrocytoma. Astrocytomas are glial tumors arising from astrocytes and occur primarily in the cerebral hemispheres, affecting roughly 15,000 individuals annually in the United States—characterized by a dismal prognosis, with 2-year and 5-year survival rates of 38.1% and 28.6%, respectively, for high-grade lesions [2].

Recent advances in cancer research have highlighted metabolic reprogramming as a hallmark of tumor progression, particularly in highly energy-demanding malignancies such as brain cancers. Tumor cells frequently exhibit profound alterations in mitochondrial metabolism, lipid utilization, and bioenergetic pathways that support rapid proliferation and survival under metabolic stress. Among the metabolic regulators implicated in these processes, L-carnitine and its derivative acetyl-L-carnitine have gained increasing attention due to their central role in mitochondrial fatty acid transport and cellular energy homeostasis [3,4]. L-carnitine functions as a key mediator of the carnitine shuttle, facilitating the transport of long-chain fatty acids across the mitochondrial membrane for β-oxidation and ATP production [5,6]. Through this mechanism, L-carnitine contributes to maintaining metabolic flexibility, regulating intracellular acetyl-CoA levels, and protecting cells against mitochondrial dysfunction and oxidative stress [3,5].

In addition to its metabolic role, increasing evidence suggests that disturbances in carnitine metabolism are associated with several pathological conditions affecting the nervous system, including neurodegenerative and psychiatric disorders. Reduced levels of L-carnitine and acetyl-L-carnitine have been reported in patients with major depressive disorder and correlate with disease severity, suggesting that alterations in mitochondrial energy metabolism may contribute to neuronal dysfunction [7,8]. Similarly, decreased concentrations of acetyl-L-carnitine and other acyl-carnitines have been observed in the progression from mild cognitive impairment to Alzheimer’s disease, supporting the hypothesis that impaired fatty-acid transport and mitochondrial metabolism may contribute to neurodegeneration [9]. Despite these findings, the role of L-carnitine in the metabolic regulation of tumor cells, particularly within the context of brain cancers, remains insufficiently characterized. Recent studies have highlighted that dysregulation of the carnitine shuttle and fatty acid β-oxidation pathways may contribute to metabolic plasticity, mitochondrial adaptation, and therapy resistance in cancer cells [10,11,12]. Furthermore, increasing evidence indicates that L-carnitine and acyl-carnitine metabolism may influence tumor bioenergetics, oxidative stress responses, and mitophagy-related pathways, suggesting a broader role of carnitine metabolism in cancer progression and metabolic reprogramming [13,14,15].

Given the growing interest in metabolic modulation in cancer, we undertook this study to evaluate the impact of L-carnitine on human brain cancer cells. L-carnitine is widely consumed as a dietary supplement to enhance energy metabolism and athletic performance, and is commonly added to energy drinks and functional foods [4,16]. Despite its widespread use, its effects on the biochemical phenotype of tumor cells remain poorly characterized. To systematically investigate these effects, we applied confocal Raman spectroscopy and imaging, enabling label-free, high-resolution mapping of cellular biochemical changes in response to L-carnitine (tartrate). This approach allows us to characterize L-carnitine-induced alterations in lipid- and protein-associated vibrational features in brain cancer cells, providing insight into its potential impact on cellular energy-related processes and biochemical homeostasis.

In biomedical research, Raman imaging provides a distinct advantage as a label-free, non-invasive method requiring minimal sample preparation, making it ideal for in situ molecular diagnostics and live-cell studies. Recent investigations highlight its ability to probe complex biological processes and disease mechanisms [17,18,19,20,21,22]. In cancer research, Raman imaging enables precise spatial mapping of biochemical changes, offering promising avenues for early diagnosis and targeted therapeutic strategies.

## 2. Materials and Methods

### 2.1. Reference Chemicals

L-Carnitine (tartrate) (36687-82-8) was obtained from Sigma-Aldrich (St. Louis, MO, USA).

### 2.2. Cell Culture and Preparation for Micro-Spectroscopy

The studies were performed on a human astrocytoma CCF-STTG1 (ATCC, CRL-1718) purchased from the American Type Culture Collection (ATCC). The CCF-STTG1 cells were maintained in RPMI1640 Medium (ATCC, no. 30-2001) supplemented with 10% fetal bovine serum (ATCC, no. 30-2020) without antibiotics in a humidified incubator at 37 °C and 5% CO_2_ atmosphere. Cells were seeded on a CaF_2_ window in a 35 mm Petri dish at a density of 5 × 10^4^ cells per Petri dish the day before the examination.

For the experiment involving L-carnitine (tartrate) treatment, CCF-STTG1 cells were incubated with 0.5 mM L-carnitine (tartrate) for 24 h. This concentration was selected to induce measurable biochemical alterations in human brain cancer cells, as assessed by confocal Raman imaging. Although 0.5 mM exceeds typical physiological plasma levels of L-carnitine (approximately 0.03–0.06 mM under basal conditions and up to ~0.1–0.2 mM following oral supplementation), it remains within the commonly applied in vitro range used to overcome limitations related to cellular uptake, compartmentalization, and metabolic turnover. In humans, oral supplementation at doses of 1–3 g/day increases circulating levels only modestly due to restricted bioavailability and efficient renal clearance, preventing systemic concentrations from approaching the millimolar range. Therefore, the selected concentration is appropriate for probing acute intracellular effects of L-carnitine, particularly its impact on mitochondrial fatty acid transport, β-oxidation flux, redox homeostasis, and lipid metabolic remodeling, thereby providing mechanistic insight into its potential role in cancer cell metabolism.

Before Raman imaging examination, cells were fixed with 4% formalin solution (neutrally buffered) for 10 min and kept in Phosphate-Buffered Saline (PBS, Gibco, no. 10010023) during the experiment.

### 2.3. Raman Data Acquisition and Analysis

Raman measurements of the human astrocytoma were conducted on a WITec confocal alpha 300 Raman microscope. The configuration of the experimental setup for 532 nm was as follows: the diameter of the fiber was 50 μm, a monochromator Acton-SP-2300i with a CCD camera Andor Newton DU970-UVB-353, a water immersion objective 40×, the laser excitation power was 10 mW, integration time of 0.3 s per pixel in enhanced mode (EMCCD). Raman images were recorded with a spatial resolution of 1 × 1 µm. A typical Raman map of a single cell consists of 2100 Raman spectra (map size 60 × 35 µm). Raman data analysis was performed using WITec (WITec Project Plus 4) and OriginPro 2024 programs. The cosmic rays were removed from each Raman spectrum (model: filter size: 2, dynamic factor: 10), and the smoothing procedure with baseline correction: Savitzky–Golay method was also implemented (model: order: 4, derivative: 0). Raman imaging data were analyzed by the Cluster Analysis method described in our previous papers [23]. Briefly, Cluster analysis is an analytical approach that groups Raman spectra into distinct sets known as clusters. Each of the clusters (the Raman spectra) must be as similar as possible in contrast to the Raman spectra belonging to another group. The separation of *n* observations (*x*) into *k* (*k*  ≤ * n*) clusters *S* should be performed to minimize the variance (*Var*) according to the formula:

$$\arg\underset{s}{\min}\sum_{i=1}^{k}\sum_{x\in Si}{\left|\left|x\mu_{i}\right|\right|}^{2}=\arg\underset{s}{\min}\sum_{i=1}^{k}|S_{i}|VarS_{i}$$

where *μ_i_* is the mean of experimental points. In our study, the number of clusters was 6. Each cluster is characterized by specific average Raman spectra, which reflect the inhomogeneous distribution of the sample. The total number of Raman spectra from Raman imaging of CCF-STTG1 cells was *n*(control) = 9900; *n*(L-carnitine (tartare)) = 10,380 across two biological replicates. In addition, principal component analysis (PCA) was performed on SNV-normalized spectra obtained from average line-scan measurements across 27 individual cells per condition (control and L-carnitine (tartare)-treated), ensuring statistically robust representation of cellular heterogeneity.

### 2.4. Statistical Analysis

The data were processed, making use of multivariate analysis. Data processing was performed using Project Plus 6.2 (WITec GmbH, Ulm, Germany) and Origin 2024 (OriginLab, Northampton, MA, USA). Chemometric analysis was performed in PLS Toolbox (EigenvectorResearch Inc., Manson, WA, USA) operating in MATLAB 2017 (MathWorks, Natick, MA, USA). Prior to multivariate modeling, the Raman spectra were preprocessed using a sequence of standardized procedures to enhance spectral quality and minimize non-chemical variance. Smoothing was applied using a 0th-order Savitzky–Golay filter with a 15-point window and polynomial interpolation of the spectral tails. Baseline correction was subsequently performed using a third-order polynomial fitted to nine user-defined spectral regions (89 reference points), followed by standard normal variate (SNV) normalization to correct for scattering-related intensity fluctuations. Principal component analysis (PCA) was conducted using the singular value decomposition (SVD) algorithm, with three principal components retained for downstream interpretation based on explained variance and model stability. Model performance and robustness were evaluated through Venetian blinds cross-validation with 10 splits and a blind thickness of 1. RMSEC (root mean square error of calibration) and RMSECV (root mean square error of cross-validation) represent reconstruction errors of the PCA model and were low (0.208 and 0.239, respectively), indicating that the selected principal components adequately capture the variance of the spectral dataset with good model stability and minimal overfitting.

## 3. Results

**Figure 1:**
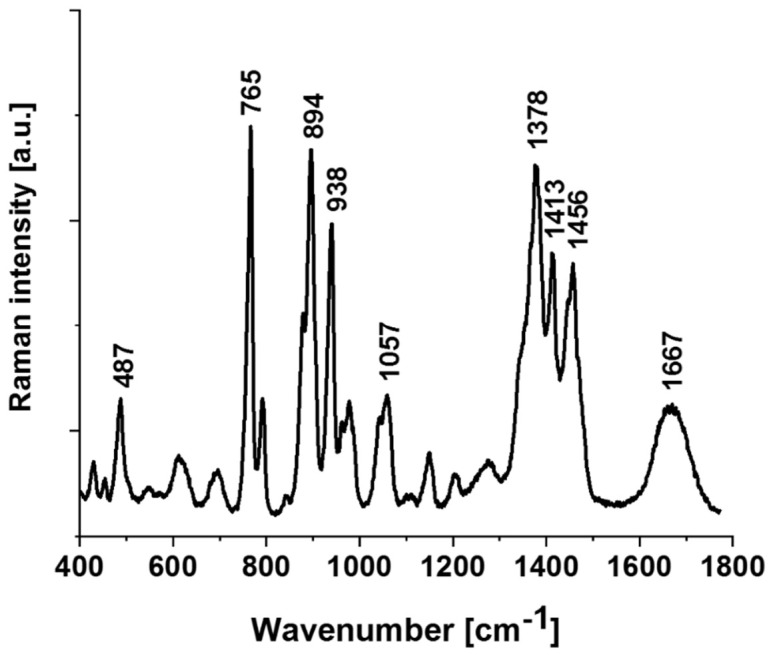
Raman spectrum of L-carnitine (tartrate).

**Figure 2:**
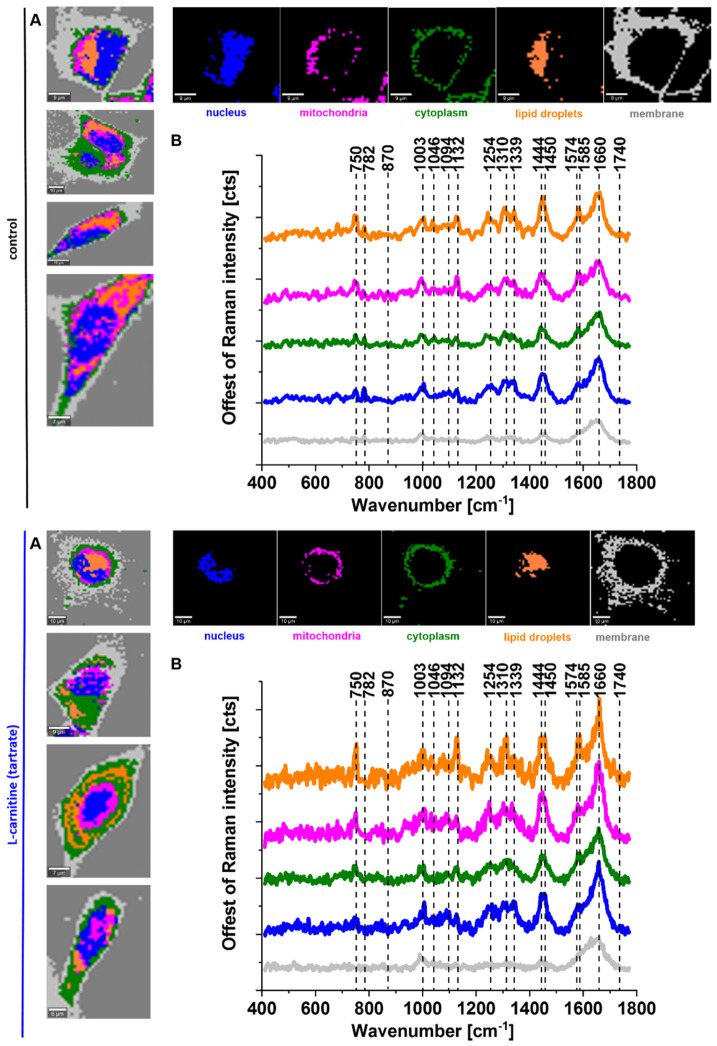
Typical Raman imaging of astrocytoma cells before and after L-carnitine (tartrate) treatment for 24 h. (**A**) Raman maps of a single astrocytoma cell before (control) and after treatment (L-carnitine (tartrate)) and (**B**) cluster spectra of cytoplasm (green), nucleus (blue), mitochondria (magenta), lipid droplets (orange), and membrane (grey). Raman imaging: 532 nm, 10 mW, 1 μm, EMCCD 0.3 s.

In this study, we investigated the biochemical alterations induced by L-carnitine exposure—administered as its tartrate salt—in human brain cancer cells using confocal Raman imaging, a label-free and non-destructive vibrational technique that enables spatially resolved characterization of major classes of biomolecules at subcellular resolution. Figure 1 displays the Raman spectrum of crystalline L-carnitine (tartrate), while Figure 2 presents representative Raman imaging maps of control human astrocytoma cells (CCF-STTG1) and cells treated with L-carnitine (tartrate).

L-carnitine (tartrate) exhibits a highly characteristic Raman fingerprint arising from the vibrational contributions of both the quaternary ammonium-containing L-carnitine moiety and the poly-hydroxylated dicarboxylate tartrate anion. The low-frequency region is dominated by skeletal deformations of the tartrate backbone, including a pronounced C–C–C/C–O–C bending mode near 487 cm^−1^. Mid-frequency signatures reflect the aliphatic chain dynamics of L-carnitine, with a CH_2_ rocking vibration at ~765 cm^−1^ and mixed C–C/C–O stretching modes between 890 and 950 cm^−1^ associated with both the alcohol groups and the N^+^(CH_3_)_3_–CH_2_ fragment. A strong C–O stretching band of the hydroxyl groups appears at ~1057 cm^−1^, while higher-frequency regions are marked by distinct contributions from the quaternary ammonium group, including the symmetric and asymmetric CH_3_ bending modes at ~1378 and ~1456 cm^−1^, respectively. The carboxylate groups generate two diagnostic vibrations: the symmetric ν_s_(COO^−^) near 1413 cm^−1^ and a broad, hydrogen-bond-sensitive antisymmetric ν_as_(COO^−^) band centered around 1667 cm^−1^.

As shown in Figure 2B, the Raman spectra display distinct vibrational bands at 750, 782, 870, 1003, 1046, 1094, 1132, 1254, 1310, 1339, 1444, 1574, 1585, 1654, 1660 and 1740 cm^−1^, corresponding to characteristic modes of lipids, proteins, carbohydrates, and nucleic acids distributed across diverse cellular compartments. Tentative vibrational assignments are summarized in Table 1. The distinct molecular composition of each organelle produces characteristic Raman signatures, enabling clear spectral discrimination. Correspondingly, the Raman imaging data (Figure 2A) resolve major cellular substructures—including the plasma membrane, nucleus, cytoplasm, mitochondria and lipid droplets—with high spatial fidelity.

**Figure 3:**
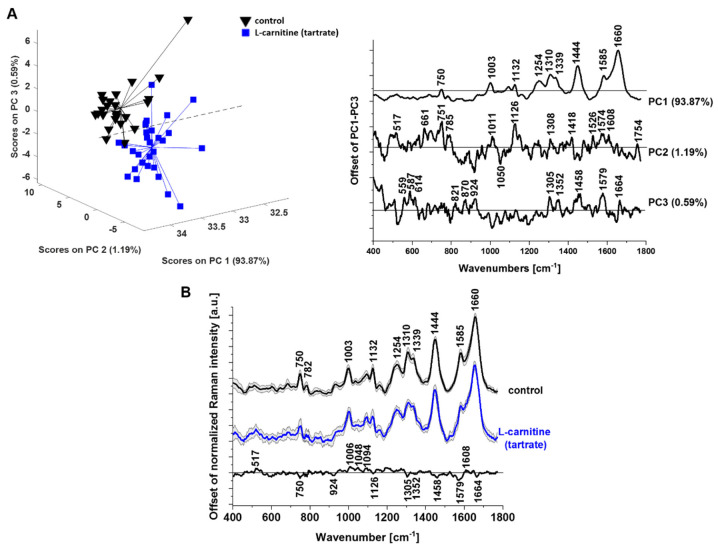
Principal component analysis of SNV-normalized Raman spectra (**A**) with mean ± standard deviation and difference Raman spectra (**B**). Shown are scatter plots of the scores for the first three principal components derived from average line-scan spectra across individual astrocytoma cells (control and treated with L-carnitine (tartrate)). The model showed low reconstruction and cross-validation errors (RMSEC = 0.208219; RMSECV = 0.238548), indicating that the selected principal components adequately capture the dataset variance with good stability and minimal overfitting. The analysis is based on datasets acquired from 27 individual cells for each experimental condition (across two biological replicates), providing a statistically robust representation of cellular heterogeneity.

Principal component analysis (PCA) was performed on SNV-normalized Raman spectra to identify the dominant sources of biochemical variance in L-carnitine (tartrate)-treated astrocytoma cells, following standard preprocessing (cosmic ray removal, baseline correction) to reduce non-biological variability and ensure chemically meaningful interpretation of the principal components (Figure 3A). The first principal component (PC1) accounted for 93.87% of the total variance and was characterized by strong loadings at 750, 1003, 1132, 1254, 1310, 1339, 1444, 1585, and 1660 cm^−1^, corresponding primarily to protein, lipid, nucleic acid, and cytochrome-associated vibrations. PC1, therefore, reflects the fundamental molecular composition of the cells. The second principal component (PC2), explaining 1.19% of the variance, displayed characteristic bands at 517, 661, 751, 785, 1011, 1126, 1308, 1418, 1526, 1574, and 1608 cm^−1^, indicating more subtle differences associated with lipid packing, nucleic acid structure, and heme-related features. PC3 captured 0.59% of the variance and exhibited loadings at 559, 587, 614, 821, 870, 924, 1305, 1352, 1458, 1579, and 1664 cm^−1^, reflecting fine variations in lipid saturation, protein conformation, and aromatic modes. Together, PC2 and PC3 capture treatment-related and subcellular biochemical heterogeneity beyond the dominant cellular signature described by PC1.

## 4. Discussion

In the principal component analysis (PCA) of the cellular Raman spectra (shown in Figure 3), PC1 accounts for the vast majority of the variance (93.87%), whereas PC2 (1.19%) and PC3 (0.59%) capture more subtle spectral changes associated with metabolic heterogeneity and L-carnitine (tartrate) supplementation. The loading profile of PC1 is dominated by bands at 750, 1003, 1132, 1254, 1310, 1339, 1444, 1585, and 1660 cm^−1^, which collectively represent the “baseline” biochemical phenotype of astrocytoma cells. The peak at ~750 is characteristic of cytochrome *c*/heme proteins vibration, while 1003 cm^−1^ corresponds to phenylalanine, and 1132–1339 cm^−1^ reflects a combination of C–C and C–N stretching in proteins and lipids, as well as nucleic acid ring modes. The strong CH_2_/CH_3_ deformation band at 1444 cm^−1^ and the Amide I/C=C band near 1660 cm^−1^ indicate that PC1 is primarily driven by the protein-lipid matrix of the cells, with contributions from mitochondrial cytochromes, thus describing the overall molecular composition rather than treatment-specific effects.

PC2 is characterized by bands at 517, 661, 751, 785, 1011, 1126, 1308, 1418, 1526, 1574, and 1608 cm^−1^, suggesting that it captures secondary sources of variance linked to more specific metabolic adjustments. The bands at 661, 751, and 785 cm^−1^ are associated with nucleic acids and heme proteins. The peaks near 1011 and 1126 cm^−1^ reflect additional protein and lipid skeletal modes, and the region around 1308 and 1418 cm^−1^ involves CH_2_/CH_3_ deformations and COO^−^ vibrations, pointing to variations in lipid packing and backbone conformation. The 1526 cm^−1^ band can be linked to C=C stretching in conjugated systems (e.g., unsaturated lipids or carotenoid-like species), whereas 1574 and 1608 cm^−1^ correspond to aromatic ring vibrations of amino acids and nucleobases. Together, this pattern indicates that PC2 likely reflects changes in mitochondrial and membrane-associated lipids, nucleic acid content, and possibly redox-active chromophores, which are consistent with metabolic remodeling upon L-carnitine treatment (e.g., altered β-oxidation and mitochondrial activity).

PC3 shows prominent loadings at 559, 587, 614, 821, 870, 924, 1305, 1352, 1458, 1579, and 1664 cm^−1^ and thus represents an even more subtle level of biochemical variability. The 559–614 cm^−1^ region can be attributed to low-frequency modes of heme-containing proteins and aromatic amino acids, while bands at 821–924 cm^−1^ are associated with C–C stretching in proteins and lipids and with carbohydrate-related vibrations. The peaks at 1305 and 1352 cm^−1^ correspond predominantly to CH_2_ twisting/wagging modes of lipids and CH deformation of proteins and nucleic acids, and the band at 1458 cm^−1^ reflects CH_2_/CH_3_ bending in membrane lipids. Finally, the features at 1579 and 1664 cm^−1^ are indicative of aromatic ring modes and Amide I/C=C vibrations, respectively. These PC3 loadings suggest fine adjustments in lipid saturation and packing, protein secondary structure, and perhaps mitochondrial versus nuclear contributions, which may distinguish subpopulations of cells with differential responses to L-carnitine (for example, cells with enhanced fatty acid oxidation and altered mitochondrial redox status versus more glycolytic phenotypes).

Taken together, PC1 describes the dominant, constitutive Raman signature of astrocytoma cells, whereas PC2 and PC3 capture treatment-related and subcellular metabolic variability. The enrichment of lipid-, protein-, nucleic acid-, and cytochrome/heme proteins-related bands in PC2 and PC3 supports the notion that L-carnitine supplementation modulates mitochondrial function and lipid metabolism, leading to detectable shifts in the Raman-derived metabolic phenotype of human brain cancer cells.

To further characterize the Raman-detectable biochemical alterations induced by L-carnitine supplementation, Pearson correlation coefficients were calculated (Table 2) to compare the average Raman cluster spectra of control and treated cells following 24 h of incubation. This quantitative analysis enabled an assessment of spectral similarity across distinct cellular compartments, including the plasma membrane, nucleus, mitochondria, cytoplasm, and lipid droplets. All differences reported in Table 2 reached statistical significance (*p* < 0.05), indicating consistent, albeit subtle, treatment-associated biochemical variations. Notably, the lowest correlation coefficient was observed for lipid droplets, suggesting relatively more pronounced alterations in these compartments in response to L-carnitine (tartrate) exposure, while overall spectral similarity remained high.

Although these findings suggest a biologically relevant role for L-carnitine in cancer metabolism, further studies are needed to define its precise molecular targets, clarify its context-dependent effects across tumor types, and evaluate its translational potential in combination therapies. In particular, as a regulator of mitochondrial fatty acid transport and β-oxidation, L-carnitine may modulate redox balance and metabolic plasticity; therefore, integrating label-free Raman imaging with transcriptomic and metabolomic profiling will be essential to obtain a spatially and mechanistically resolved understanding of its impact in oncological contexts.

Future studies would benefit from integration with complementary molecular biology approaches, such as qPCR and Western blotting, to further substantiate the Raman-based findings. Expanding the number of analyzed cells would also strengthen the statistical power and generalizability of the results. Accordingly, the present findings should be interpreted with appropriate caution and considered as preliminary. Notwithstanding these limitations, the study demonstrates the considerable potential of Raman imaging to probe the effects of L-carnitine (tartrate) in human astrocytoma cells (Grade IV).

## 5. Conclusions

In summary, our findings demonstrate that confocal Raman spectroscopy offers a sensitive, label-free approach for mapping the biochemical effects of L-carnitine (tartrate) in human astrocytoma cells (Grade IV). L-carnitine (tartrate) supplementation induced discernible alterations in lipid, protein, nucleic acid, and cytochrome-associated vibrational signatures, which might reflect its role in modulating mitochondrial activity and cellular energy metabolism. PCA confirmed that even subtle, treatment-dependent spectral shifts—captured primarily in PC2 and PC3—are associated with alterations in lipid-related and redox-sensitive biochemical features. These results indicate that Raman imaging can resolve treatment-dependent biochemical heterogeneity at the single-cell level, offering insight into cellular responses to exogenous metabolic modulators. Overall, this work highlights the potential of vibrational spectroscopy as a promising approach with translational relevance for assessing biochemical responses in cancer cells and for evaluating the cellular impact of widely consumed dietary supplements such as L-carnitine (tartrate).

## Figures and Tables

**Table 1:** Tentative assignments of Raman bands [17,18,24,25,26].

Wavenumbers [cm^−1^]	Tentative Assignments
487	skeletal deformations of the tartrate backbone, C–C–C/C–O–C bending mode, L-carnitine (tartrate)
750	out-of-plane vibrations of the porphyrin ring, cytochrome *c*/heme proteins
765	CH_2_ rocking vibration, L-carnitine (tartrate)
782–789	cytosine, uracil, thymine, pyrimidine bases, ring breathing modes
845–870	lactate
890–950	mixed C–C/C–O stretching modes, L-carnitine (tartrate)
1002–1004	phenylalanine, proline, symmetric stretching (ring breathing) mode of phenyl group
1046	C–C stretching, proteins
1057	C–O stretching band of the hydroxyl groups, L-carnitine (tartrate)
1094	PO_2_^−^, symmetric stretching mode of phosphate esters, DNA/RNA
1126	C–H deformation and in-plane porphyrin breathing, cytochrome *c*/heme proteins
1168–1174	C–C_6_H_5_ phenylalanine, tryptophan
1250–1350	extended Amide III, coupled C–H, N–H deformation modes, peptide backbone
1250–1260	=CH deformation, lipids
1300–1306	CH_2_ twist, lipids
1310	vibrations of C_m_–H mode, cytochrome *c*/heme proteins
1332–1339	adenine/guanine, nucleic acids
1378	quaternary ammonium group, the symmetric CH_3_ bending, L-carnitine (tartrate)
1413	carboxylate groups, symmetric ν_s_(COO^−^), L-carnitine (tartrate)
1425–1475	CH_2_ and CH_3_ deformations, antisymmetric methyl and methylene deformations, peptide side chains, phospholipids
1456	quaternary ammonium group, asymmetric CH_3_ bending, L-carnitine (tartrate)
1574	C=C stretching, purine bases, DNA/RNA
1585	C_a_–C_m_ stretching within the conjugated porphyrin (methine bridge stretching), cytochrome *c*/heme proteins
1654	Amide I, C=O stretching mode, peptide linkage
1660	C=C stretching, lipids
1667	carboxylate groups, hydrogen-bond-sensitive antisymmetric ν_as_(COO^−^), L-carnitine (tartrate)
1740–1746	C=O stretching, ester group of lipids and phospholipids

**Table 2:** Pearson correlation coefficients obtained for the comparison of average Raman cluster spectra between control and L-carnitine (tartrate)—treated cells after 24 h of incubation, across membrane, nucleus, mitochondria, cytoplasm and lipid droplets.

	Membrane	Nucleus	Mitochondria	Cytoplasm	Lipid Droplets
control vs. L-carnitine (tartrate)	0.9767	0.9830	0.9722	0.9777	0.8889

## Data Availability

The datasets generated and analyzed during the current study are available from the corresponding author on reasonable request.
